# Integration of high-resolution imaging through scattering medium into a disposable micro-endoscope via projection of 2D spots-array

**DOI:** 10.1038/s41598-023-46657-0

**Published:** 2023-11-13

**Authors:** Shimon Elkabetz, Oran Herman, Amihai Meiri, Asaf Shahmoon, Zeev Zalevsky

**Affiliations:** 1 Faculty of Engineering, Bar Ilan University, 5290002 Ramat Gan, Israel; 2 Zsquare Ltd, 43 Hasivim St., 4959501 Petah Tikva, Israel

**Keywords:** Biophysics, Optics and photonics

## Abstract

The objective of this research includes integration of high-resolution imaging through scattering medium, such as blood, into a disposable micro-endoscope. A fiber laser integrated into the micro-endoscope as part of its illumination channel, allows to project a tunable array of spots of light onto an object, that is located behind the scattering medium. We have a laser fiber as part of the illumination channel of a disposable micro-endoscope. By using proper optics, we convert the temporal modulation of the laser into spatial distribution. Thus, the result is generation of spatial spots when using a pulsed laser. The detection channel is a holographic recording of the collected back scattered light, that allows extraction of the electrical field. By time integrating the field we obtain the realization of the spatial array of illumination spots formed on top of the inspected object and behind the scattering medium. By changing the temporal modulation of the illumination laser (changing its temporal photonic signals), we can tune the positions of the spots in the illumination array. If the distance between the projected spots is larger than the imaging resolution, then by applying localization microscopy algorithms together with scanning of the position of the spots in the array, will yield a high-resolution reconstruction of the inspected object. We theoretically and experimentally demonstrate the discussed operation principle and show the potential of the proposed concept as a modality in medical endoscopic procedures.

## Introduction

Having high imaging resolution is one of the most important tasks that companies of micro-endoscopy aim to achieve^1^. However, since micro-endoscopes are designed for medical procedures which usually involve blood, the real limiting factor for the imaging resolution is not the inherent capabilities of the imager but rather in many cases, the working conditions of highly light scattering environment involving blood^2^.

In the last 60 years, many researchers have paved the way for developing technologies to allow imaging through a scattering media^3–6^. Therefore, imaging through blood, which is being highly scattering medium, is a technologically complicated task. While various recent concepts were previously demonstrated^7–10^, only a few are suitable to be implemented in micro-endoscopy since they do not require a calibration beacon to be placed behind the scattering medium neither need to physically model the scattering medium itself^11–13^.

In our previous publication, we showed a novel approach allowing to image through a scattering medium by enhancing the signal to noise ratio (SNR) of the information photons coming from the plane of the inspected object while attenuating the defocused noise photons related to scattering which are responsible for the image resolution degradation^14^. This was obtained by using a special array of diffractive optical elements (DOEs) integrated into the micro-endoscope handle which is constructed from an array of micro annular like elements that aim to block the defocused photons and transmit the focused one. Because the imaging concept involves the usage of an array of elements it also assumes spatial discretization of the object to be imaged. The object also needs to be constructed from an array of information points. In order to fulfill this requirement, we illuminate the inspected object through the scattering medium, with an array of spots.

In this paper, we present a novel idea of how to project such a tunable array of illuminating spots that will illuminate the inspected object positioned behind the scattering medium. The idea is proven mathematically and then experimentally validated. Its incorporation with the DOEs array-based imaging concept of Ref.^13^, opened the path of new directions in high resolution imaging capabilities for micro-endoscopy. The proposed novel idea includes a time modulated laser-based illumination channel and a holographic imaging module. Both could be miniaturized and integrated as part of the micro-endoscopy handle.

## Materials

**Figure 1:**
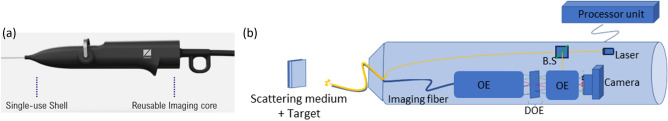
The developed single use fiber-based micro-endoscope.

Previously presented a single use micro-endoscope is imaged in Fig. 1a. This developed novel micro-endoscopy platform is a novel device^15^ and it is based on two main parts: (1) The single-use shell which includes the shell that involves the imaging and the illumination fibers. The imaging fiber is a multi-core ultra-thin fiber (having an external diameter of only about 450 microns) and the illumination fiber is a conventional multi-mode fiber. (2) The reusable imaging core which includes the camera and most of the imaging lenses allows implementing the scheme that is shown in this article in Fig. 1b.

**Figure 2:**
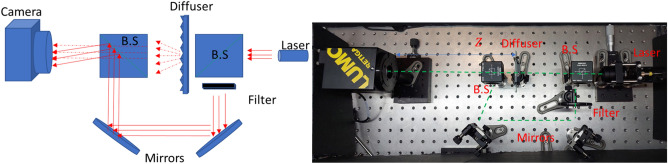
Setup for projection of an array of discrete points. On the left side is the schematic sketch of the constructed experimental setup and on the right is its image from the lab.

In this paper, we present an optical bench constructed novel experimental setup that later on is to be integrated into the handle of the micro-endoscope of Fig. 1b. The constructed optical setup is demonstrated in Fig. 2 and it involves a green laser at a wavelength of 532nm and a camera (Lumo Retiga with a 2688 $\times$ 2200 pixels array and 4.54 $\times$ 4.54 ${\upmu {\rm m}}$ pixel size) working in holographic recording mode, i.e., the illumination laser beam that illuminates the object though the scattering medium is also used as a reference beam to illuminate the camera directly in order to allow recording of the optical field (as done in off-axis holography^16^) and not optical intensity (like in regular imagers). In the schematic sketch and the optical setup of Fig. 2 the aim is to use a pulsed laser to move the position of the illumination spot relative to the diffuser (imitates the scattering medium) to create a scanning pattern, an array of spots is generated on top of the detector (that is positioned where the object should be positioned). Hence, the experiment procedure involves a moving laser on one axis along the diffuser plane. Using a triggering mode of the camera, we acquired 20 images in the far-field range. The holographic recording mode allowed us to extract the field data of each image, and finally, we summed this data to obtain the spatial array of illumination spots.

It is important to note that considering the camera sensitivity and the absorption coefficient of human blood tissue/mucous, for 830 nm is 0.05 ${\mathrm{cm}}^{-1}$, it is likely that tens of milliwatts of laser power will be sufficient to obtain a clear image. The typical maximum power ENT in vivo applications is $\approx 100 \mathrm{mW}$. In our experiment, we used a laser power range of 1–5 mW.

## Method

In this section, we mathematically derive the proposed concept and prove the idea behind it. We will start for simplicity with the 1D case and then show the 2D case.

### One dimensional case

Let us assume that we illuminate the scattering medium with time changing pattern D(x,t). This pattern actually is a spot of light that its location and amplitude is changed in time:

1 $$D\left(x,t\right)=a\left(t\right) rect\left(\frac{x-vt}{\Delta x}\right)$$

where $v$ is the shifting velocity for the center of the illuminating spot D(x,t) and a(t) is its time changing amplitude of the illumination (so the amplitude of the illumination laser is modulated in time).

We assume that we illuminate with a laser beam (spatially and temporally coherent illumination). If the shifts are small, the speckle pattern that is generated behind the scattering medium has the memory effect^17^ and it will mainly shift as much as the illumination spot, rather than change. We will denote the speckle pattern generated behind the scattering medium due to our illumination spot $rect\left(\frac{x-vt}{\Delta x}\right)$ as: $\mathrm{s}(\mathrm{x}-v\mathrm{t})$. Thus, the time modulated illumination of $a(t)rect\left(\frac{x-vt}{\Delta x}\right)$ can be expressed as $a(t)\mathrm{s}(\mathrm{x}-v\mathrm{t})$.

In the imaging camera a time integration is performed and thus the overall projected speckle pattern that is generated behind the scattering medium will be:

2 $$\int \mathrm{a}(\mathrm{t})\mathrm{s}(\mathrm{x}-v\mathrm{t})dt$$

Note that since the detection is done via digital holography, one can separate capture the term of the temporal integration over the optical field (and not its intensity).

We will assume that those are not fully developed speckles and that the object is located in a distance from the scattering medium and thus the pattern P(x) that is projected on the object (located behind the scattering medium) is the spatial Fourier transform of the above-described speckle pattern^18^:

3 $$P\left(x\right)=\int \left(\int \mathrm{a}(\mathrm{t})\mathrm{s}({{\rm x}{^\prime}}-v\mathrm{t})dt\right)exp\left(\frac{-2\pi ix{^\prime}x}{\lambda Z}\right)dx{^\prime}$$

Where Z is the distance between the scattering medium and the object that we aim to image and $\lambda$ is the optical wavelength and in Eq. (3) we performed the optical Fourier transform or the far field Fresnel approximation over Eq. (2). The last equation can be written as:

4 $$P\left(x\right)=S\left(\frac{x}{\lambda Z}\right)\left(\int \mathrm{a}(\mathrm{t})exp\left(\frac{-2\pi ivtx}{\lambda Z}\right)dt\right)$$

Where $S\left(\frac{x}{\lambda Z}\right)$ is the spatial Fourier transform of s(x) and it is the fully developed speckle pattern (the far field of the not fully developed speckle pattern). Note that what we have obtained in Eq. (4) is that the expression which multiplies $S\left(\frac{x}{\lambda Z}\right)$ is the temporal Fourier transform of a(t) (the time changing amplitude of our illuminating spot). In addition, it is important to note that the developed endoscope is mainly aimed for ENT applications where such a scenario like in Eq. (3) is very common. The scattering medium many times is related to an infection related liquids while the imaging is made on objects positioned behind the infection without contacting it.

We will generate temporally pulsed illumination with our laser. Let us assume that the amplitude modulation equals to:

5 $$a\left(t\right)=\sum_{n}\delta \left(t-n\Delta t\right)$$

Where $\delta \left(t\right)$ is Dirac delta function, $\Delta t$ is the time duration of the pulsed illumination and n is the index of the laser pulse. According to Fourier transform properties one obtains:

6 $$\int \mathrm{a}(\mathrm{t})exp\left(\frac{-2\pi ivtx}{\lambda Z}\right)dt=\sum_{n}\delta \left(\frac{xv}{\lambda Z}-\frac{n}{\Delta t}\right)=\sum_{n}\delta \left(x-n\frac{\lambda Z}{v\Delta t}\right)$$

Thus, the final result for the pattern illuminating the inspected object becomes:

7 $$P\left(x\right)=S\left(\frac{x}{\lambda Z}\right)\sum_{n}\delta \left(\frac{x}{\lambda Z}-\frac{n}{v\Delta t}\right)$$

This illumination is obtained behind the scattering medium and this means that the projected speckle pattern is discretized by its multiplication with the discrete pattern of an array of delta spots: $\sum_{n}\delta \left(\frac{x}{\lambda Z}-\frac{n}{v\Delta t}\right)$.

It is important to note that the both the pulsed illumination a(t) as well as the speed of the spatial shift of the center of the illuminating spot, should be such that the full cycle of temporal modulation will be accomplished per the integration time of the imaging camera. For instance, if the imaging camera produces 50 frames per second (20ms per image) and we wish to generate a projection of an array of 1000 $\times$ 1000 spots, we will need to have temporal period $\Delta t$ of 20nsec per the illuminating pulses.

It is important to note that the projected array of discrete spots can be used for super resolved imaging. If the distance between the spots in the array is larger than the blurring point spread function (PSF) of the imaging lens, then by scanning with the array over the spatial area between the spots, can produce super resolved imaging with resolution equivalent to the size of each spot in the array, similarly to what is done in localization microscopy^19–21^. Scanning and changing the position of the spots in the array can be obtained as following:

8 $$a\left(t\right)=\sum_{n}exp\left(-2\pi i\beta t\right)\delta \left(t-n\Delta t\right)$$

where the parameter $\beta$ will produce the amount of spatial scanning since this proposed function for the time modulation of the laser amplitude will yield:

9 $$\int \mathrm{a}(\mathrm{t})exp\left(\frac{-2\pi ivtx}{\lambda Z}\right)dt=\sum_{n}\delta \left(t-n\Delta t\right)exp\left(-2\pi it\left(\frac{vx}{\lambda Z}+\beta \right)\right)dt=\sum_{n}\delta \left(x-n\frac{\lambda Z}{v\Delta t}+\beta \frac{\lambda Z}{v}\right)$$

Another important application for the proposed novel concept could be to generate a focusing spot behind the scattering medium while such point can be made tunable in its transversal location. For this case we will perform temporal modulation for the illuminating laser’s amplitude as:

10 $$a\left(t\right)=exp(2\pi i\beta t)$$

Which will lead to:

11 $$\int \mathrm{a}(\mathrm{t})exp\left(\frac{-2\pi ivtx}{\lambda Z}\right)dt=\delta \left(x-\frac{\beta \lambda Z}{v}\right)$$

By tunning the parameter $\beta$ we can tune the position of the focus behind the scattering medium, because mathematically speaking the delta function $\delta \left(x-\frac{\beta \lambda Z}{v}\right)$ is a focus obtained at coordinate $x=\frac{\beta \lambda Z}{v}$.

### Two-dimensional discrete case

Let us now show the generation of a 2D array of spots. In this case the movement of the illumination laser source will be in 2D. Let us assume that for shifting the projected pattern $\mathrm{s}(\mathrm{x}-\mathrm{vt})$ we will use a step motor. This means that the shifting is to be performed in discrete steps. Thus, we will have the 2D discrete version of motion:

12 $${a}_{nm}s(x+\Delta x,y+m\Delta y)$$

In the far field one will obtain a 2D discrete Fourier transform:

13 $$\sum_{n}\sum_{m}{a}_{nm}S\left(\frac{x{^\prime}}{\lambda Z},\frac{y{^\prime}}{\lambda Z}\right)exp\left(\frac{-2\pi i\Delta x}{\lambda Z}nx{^\prime}\right)exp\left(\frac{-2\pi i\Delta y}{\lambda Z}my{^\prime}\right)$$

The last expression equals to:

14 $$S\left(\frac{x{^\prime}}{\lambda Z},\frac{y{^\prime}}{\lambda Z}\right)\sum_{n}\sum_{m}{a}_{nm}exp\left(\frac{-2\pi i\Delta x}{\lambda Z}nx{^\prime}\right)exp\left(\frac{-2\pi i\Delta y}{\lambda Z}my{^\prime}\right)$$

As before we will choose temporally changing amplitude modulation to obtain a 2D array of spots (right wing of Eq. 15):

15 $$\sum_{n}\sum_{m}{a}_{nm}exp\left(\frac{-2\pi i\Delta x}{\lambda Z}nx{^\prime}\right)exp\left(\frac{-2\pi i\Delta y}{\lambda Z}my{^\prime}\right)=\sum_{n}\sum_{m}\delta \left({x}{^\prime}-n\Delta {x}{^\prime},{y}{^\prime}-m\Delta {y}{^\prime}\right)$$

Which is obtained for:

16 $${a}_{nm}=1 \Delta {x}{^\prime}=\frac{\lambda Z}{\Delta x}, \Delta {y}{^\prime}=\frac{\lambda Z}{\Delta y}$$

## Results

We will now present an experimental lab demonstration for our novel design and demonstrate the generation of the projected array of spots being formed on top of the object that is positioned behind the scattering medium.

Note that a holographic recording setup is needed for our configuration because as mathematically shown, the formation of the illuminating array of spots on the object is obtained when in the camera a time-integration of the electrical field (and not intensity) is performed.

**Figure 3:**
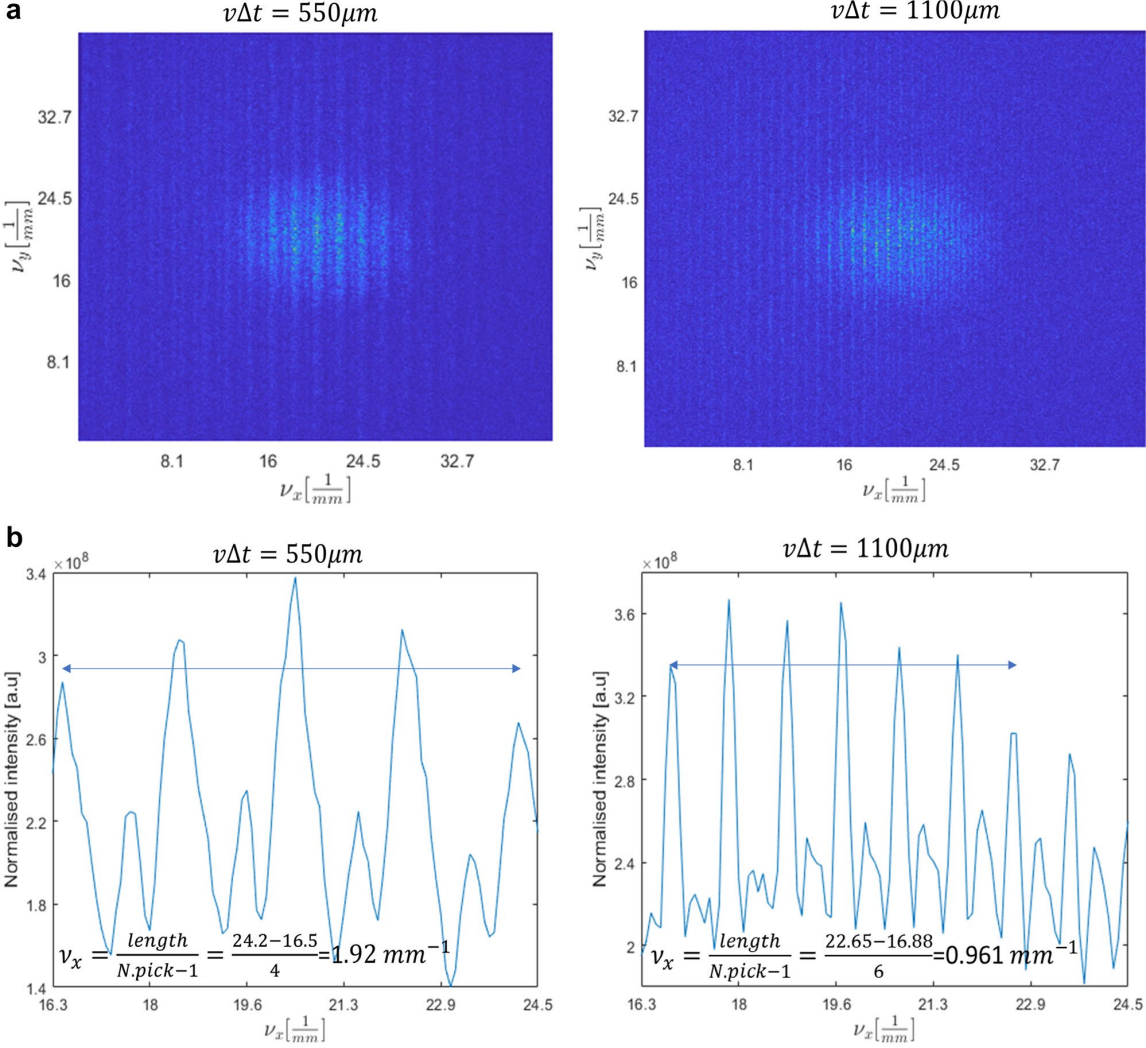
Experimental results. (**a**) Generation of projected discrete lines obtained for two different $v\Delta t$ values. (**b**) The horizontal cross of the images of (**a**).

In Fig. 3 we show the obtained experimental results. The shift was done in 1D and thus the expected projected discretization is also obtained in 1D which means that we obtain a generation of projected lines. This can clearly be seen in Fig. 3a. In Fig. 3a we presented two examples of different $v\Delta t$ values, which are inversely proportional to the illumination spatial frequency. In Fig. 3b we plot the cross section along the horizontal axis of Fig. 3a to see that the density of the generated lines matches the theory behind the proposed idea. Indeed, the spatial density of the generated projected lines matches the density expected from the theory according to the applied $v\Delta t$ values. For example, for the spatial frequency illumination of $\frac{1}{v\Delta t}=0.9 [{\mathrm{mm}}^{-1}]$, where $v\Delta t=1100 {\upmu {\rm m}}$, the spatial frequency of the generated projected lines is equal to ${v}_{x}=0.96 [{\mathrm{mm}}^{-1}]$.

Note that in Fig. 3 we presented two examples of different $v\Delta t$ values but the experiment was performed with many different $v\Delta t$ values. Table 1 summarizes the various results for such different values.

**Table 1:**
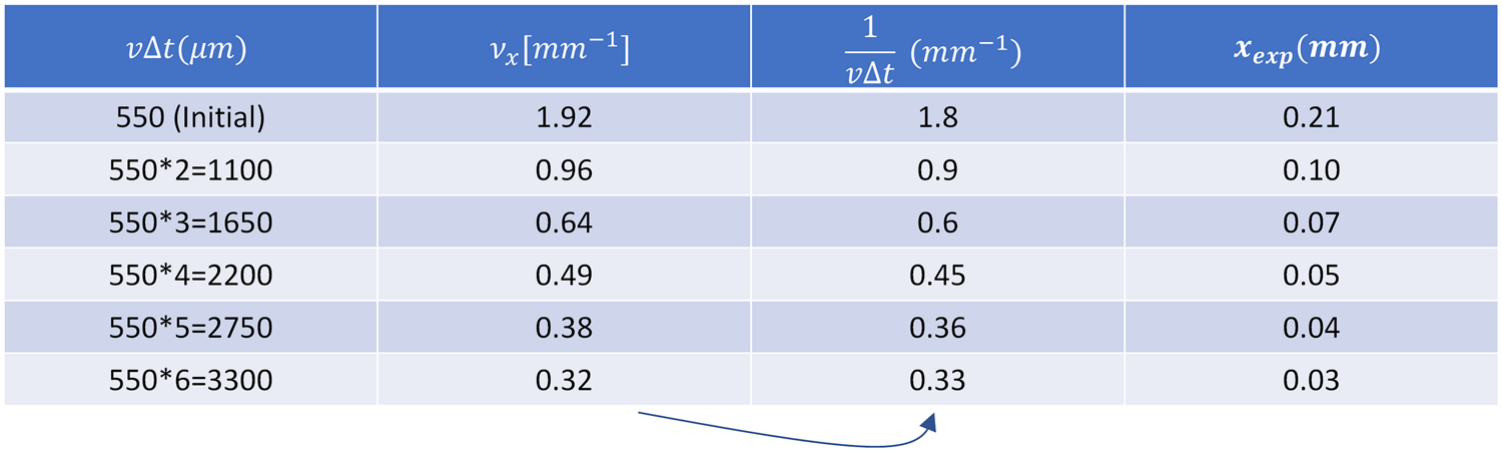
Summary of experimental results performed for different $v\Delta t$ values.

Using Eq. (7) we see a great agreement between the theoretical calculation and experimental results $\left({\nu }_{x}=\frac{1}{v\Delta t}\right)$ when calculating the value of $v\Delta t$ and comparing it with the spatial frequency ${\nu }_{x}$ that was experimentally obtained. The translation between the experimentally measured ${\nu }_{x}$ and the distance between the projected points was done according to Eq. (7) as follows:

17 $${x}_{exp}=\frac{\lambda Z}{v\Delta t}={\nu }_{x}\lambda Z$$

In the experiment our wavelength was $\lambda =532 \mathrm{nm}$ and the free space distance was Z = 200 mm.

**Figure 4:**
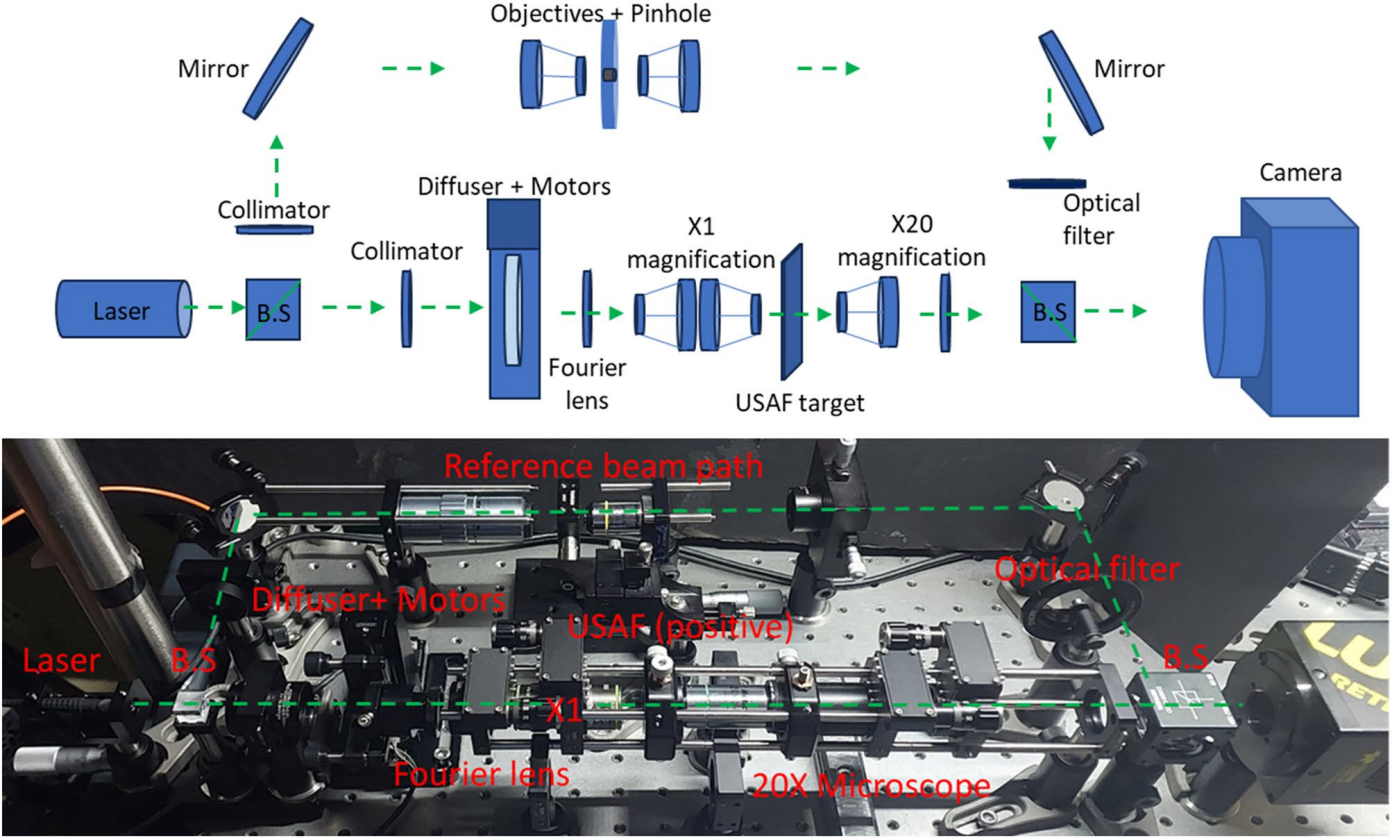
The experimental setup used for the 2D experiments.

For the 2D case, the experimental setup of Fig. 4 was constructed. It is based on the initial setup of Fig. 2, but controlled step-motors were added and in addition instead of free space propagation, the far field approximation was obtained by placing a Fourier transforming lens. In the setup, we had an optical array containing a 532nm laser source whose Illumination is split by a beam splitter into equal beams, one for the signal and the other for the reference (needed for the holographic recording). The signal beam is passing through a diffuser (scattering medium) driven by motors in the perpendicular plan. An USAF 1951 resolution target was our signal and was used as our object that was imaged by our camera. The other path of the reference passes through a 50 ${\upmu {\rm m}}$ pinhole and is reflected to the camera.

**Figure 5:**
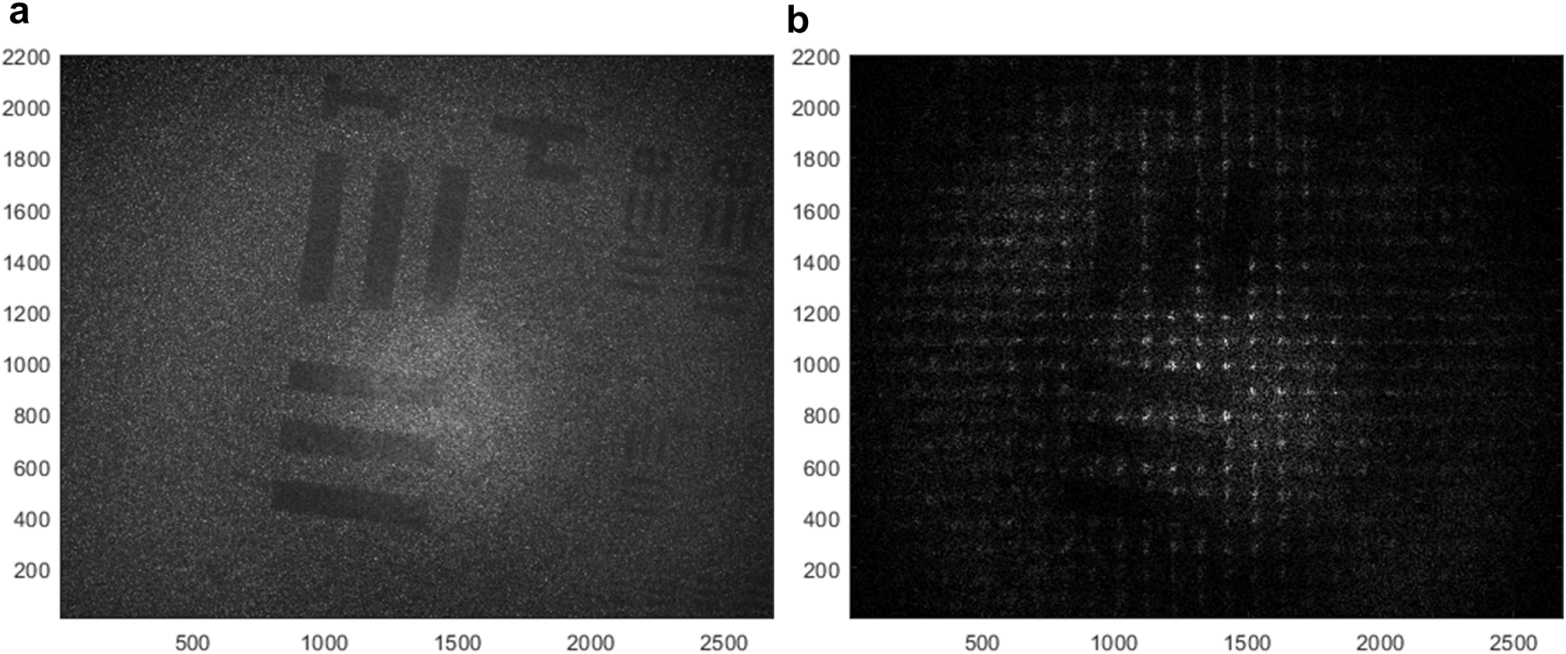
2D experimental results for projection of an array of spots through scattering medium. (**a**) A single image from 64 images matrix captured during the pojection process. (**b**) Dots pattern generated on top of an USAF 1951 (30 ${\upmu {\rm m}}$ line width) resolution target.

The obtained results for the 2D case are seen in Fig. 5. In the experiment, 64 optical field images were collected in the holographic recording done by our camera during the 2D scanning done with the step-motors. In Fig. 5a a single image from those 64 images matrix captured during the projection process, is shown. In Fig. 5b we show the obtained results where an array of dots pattern is formed (projected) on top of the USAF 1951 (30 ${\upmu {\rm m}}$ line width) resolution target while the projection is done through a scattering medium (a diffuser).

In this paper, we present two setups that demonstrate the realization of the novel idea of the paper. The difference between the two setups is that the setup of Fig. 2 is a preliminary system that presents the basics of this paper's idea without any imaged object. In addition, it shows a 1D experiment of the continuous case when the laser spot moves along the diffuser.

On the other hand, the setup demonstrated in Fig. 4 shows the concept of the 2D discrete case by using step motors. The results display a 2D spatial array of illumination spots on an imaged object as required in the final product. We can see that the setup of Fig. 4 includes many optical elements that allow us to demonstrate controlling a suitable resolution for a realistic endoscopy procedure. In addition, this setup is the closest to the final see-through blood configuration^14^.

## Discussions and conclusions

In this paper, we presented a novel time multiplexing super resolving holographic imaging concept that is designed to be integrated into a disposable micro-endoscope and which aims to allow obtaining a high-resolution imaging of objects through scattering medium such as blood.

The proposed idea is to perform imaging via a specially designed imaging channel containing an array of DOEs that significantly attenuates defocused noise photons. The imaging capability of this novel concept was previously demonstrated experimentally, but for the case where a discrete type of objects were imaged and the object’s discretization (its pixels) was matched to the number of the elements in the DOEs array.

In this paper we theoretically and experimentally demonstrate how the discussed operation principle can be used for imagining of non-discretized objects and thus be suitable for real case scenario of endoscopic imaging challenges. The novel idea presented in this paper includes laser-based illumination of the inspected object, done though the scattering medium, while generating an array of illuminating spots on top of it. That way the discretization is obtained via the illumination module instead of having any restrictions about the object itself.

The advantage resulting from the great match with the analytical calculations illustrates to us the simplicity of controlling the density of the discrete points and easy adjustment to the requirements of the endoscope DOE array. In addition, the ability to determine the resolution according to the needs is obtained without additional optical elements.

The projection of an array of illuminating spots is obtained by temporally modulating the illumination laser while slightly moving it and all this is performed when a holographic detection (senses the optical field) is done while performing time integration. This technological novelty has large potential as an important modality in medical endoscopic procedures that will in the future allow performing high resolution imaging in a medical procedure in the presence of blood despite its light scattering characteristics. Another technological advantage is the ability to distinguish an object located a few millimeters behind human tissue. It is revolutionary in every aspect and effective for many medical procedures.

## Data Availability

The datasets used and/or analyzed during the current study available from the corresponding author on reasonable request.
